# A cost comparison between patients undergoing robotic colorectal surgery with and without a clinical pathway

**DOI:** 10.1186/s12962-026-00770-9

**Published:** 2026-05-23

**Authors:** Alfonso Valenzuela Hurtado, Onur Bayram, Jörg Kleeff, Johannes Klose, Manuela De Allegri, Ulrich Ronellenfitsch

**Affiliations:** 1 Heidelberg Institute of Global Health, Heidelberg University Hospital and Faculty of Medicine, Heidelberg University, Heidelberg, Germany; 2 Department of Visceral, Vascular and Endocrine Surgery, Department of Visceral, Vascular and Endocrine Surgery, University Medical Center Halle (Saale), Martin-Luther-University Halle-Wittenberg, Ernst-Grube-Straße 40, 06120 Halle (Saale), Germany; 3 Department of General, Visceral, and Transplantation Surgery, Heidelberg University Hospital and Faculty of Medicine, Heidelberg University, Heidelberg, Germany

**Keywords:** Clinical pathway, Robotic surgery, Colorectal surgery, Micro-costing

## Abstract

**Background:**

Clinical pathways (CPs) are guidelines to standardize processes, improve quality of care and maximize resources through the implementation of evidence-based care for a medical procedure, thus reducing variation and risk. Our aim was to evaluate the cost of care for robotic colorectal surgery using a CP compared to surgery without a CP.

**Methods:**

This was a non-interventional micro-costing study with a hospital-level perspective. It was carried out using the three standard steps. First, all resources involved in the implementation of the CP were identified. Second, resource consumption was measured. Third, the resources identified were valued and measured to estimate the average cost per group. This included the costs related to perioperative care and treatment of complications. Finally, a sensitivity analysis was conducted based on the hypothetical capacity utilization of the robotic equipment, costs of CP development and a discount rate of 3% over a period of five years.

**Results:**

The study population comprised 63 patients: 21 in the CP group (robotic colorectal surgery with CP) and 42 in the no-CP group (robotic colorectal surgery without CP). The mean cost per patient in the CP group was €12,663 (range: €8,093 to €26,475), whereas in the no-CP group it was €11,128 (range: €8,357 to €17,268). The main cost drivers in the CP group were materials and disposables (33% of total costs) and staff (20% of total costs). In the no-CP group, they accounted for 38% and 20% of total costs, respectively. The CP group was used for the sensitivity analysis. In year 1, at 100% utilization the mean cost per patient was €12,364, increasing to €17,883 at 20% utilization. In year 5, at 100% utilization the mean cost per patient was €13,986, rising to €20,519 at 20% utilization.

**Conclusion:**

The evidence from our study indicates that using a CP for perioperative care of patients undergoing robotic colorectal surgery was not associated with a statistically significant reduction in cost of care.

**Supplementary Information:**

The online version contains supplementary material available at 10.1186/s12962-026-00770-9.

## Introduction

Colorectal surgery encompasses a range of techniques aimed at addressing conditions such as cancers, diverticulitis, and bowel obstruction, among others. These interventions can be carried out through open or minimally-invasive methods [1, 2]. Over the past twenty years, there has been a noticeable shift towards favoring minimally invasive approaches, now transitioning from laparoscopic to robotic surgery [1–3].

Scientific literature presents arguments both in support of and against the implementation of robotic surgery [2–5]. The robotic approach provides specific benefits in scenarios where technical challenges are prominent, thanks to its surgical precision and capabilities [6]. This is expected to result in reduced tissue trauma, decreased intraoperative blood loss, and faster postoperative recovery [6]. While there is evidence supporting its safety and efficacy, a clear advantage that justifies its considerable expense, significant learning curves and longer duration in surgery compared to laparoscopic or open colorectal surgery still needs to be demonstrated [6].

Clinical pathways (CPs) are tools that are frequently used to streamline care and improve clinical processes [7–10]. Typically, a CP is designed as a grid of tasks integrated in a timeframe, which harmonizes and streamlines the treatment plan, engaging multiple healthcare professionals involved in the patient’s care to ensure effective management [11–13]. The potential benefits of a CP are expected to lead to better outcomes by delineating various aspects, including nutritional guidance, anticoagulation measures, fluid and pain control, mobilization directives, and discharge arrangements [14–16]. Besides aiding in achieving other benefits, CPs have been proposed as a method to optimize resource allocation in a healthcare landscape characterized by escalating costs [17–19].

This study assessed the cost of care for robotic colorectal surgery using a clinical pathway (CP) compared to robotic colorectal surgery under routine clinical practice (i.e., no-CP) at a single center, the University Hospital of the Martin-Luther-University Halle-Wittenberg. The motivation for the study was to evaluate the cost of care in relation to a recently implemented CP for robotic colorectal surgery.

## Methods

### Study setting and intervention

The study was carried out in the Department of Visceral, Vascular and Endocrine Surgery at the University Hospital Halle (Saale), Martin Luther University Halle-Wittenberg, Germany. The hospital is a tertiary level facility, with 1,019 beds and 30 departments [20].

A surgical robot has been used for colorectal surgery in the Department since 2018. A multidisciplinary team of experts from the Department designed custom-made CPs for robotic colorectal surgery, one for colectomies and one for rectal resections, with the goal of standardizing and improving patient care along the continuum of treatment. The CPs were grounded in proven, evidence-based practices [21, 22]. CPs were implemented into clinical routine starting July 2021. Concurrently, a clinical study was conducted to assess the impact of CP on process and outcome quality indicators and on the cost of care in robotic colorectal surgery.

In the CP, every step necessary for standardized patient care is meticulously outlined in a structured framework, organized along a time axis. This encompasses pre-admission procedures and inpatient care from admission to discharge, including the day of surgery. The CP outlines all diagnostic and therapeutic measures to be carried out, encompassing, among others, patient monitoring, anesthesia, bowel preparation, medication, transfusions, pain management, quality assurance and documentation, patient education, and nursing tasks. The complete CP can be found in the supplementary material.

### Study design

This bottom-up micro-costing study adopted a single-center perspective and was conducted as an ancillary analysis within an ongoing study on clinical outcomes of robotic colorectal surgery with and without CP. Two groups were compared: the no-CP group, patients who underwent robotic colorectal surgery without CP (2018–2020, 42 consecutive patients), and the CP group, patients who underwent robotic colorectal surgery with CP (2021–2022, 21 consecutive patients). Patients were included consecutively before and after CP implementation, and no randomization was performed. The temporal separation between groups partly coincided with the COVID-19 pandemic, which may have influenced healthcare delivery, patient selection, and resource utilization independently of CP implementation. The costs associated with the perioperative continuum of care, i.e., the resource consumption of each patient, were calculated. This enabled us to estimate the total cost per patient and assess how CP use influences the cost of care in robotic colorectal surgery.

### Micro-costing

Our micro-costing study was carried out following the three standard steps of costing [23], which served to identify, measure, and value resources used by the patients in both the control and the CP group. As a first step, the cost items required by robotic colorectal surgery patients along the perioperative continuum of care were identified by obtaining information from the hospital’s Health Management Information System (HMIS), complemented by direct on-site observation. Subsequently, these cost items were classified into variable costs (staff, personal protective equipment, laboratory and diagnostics, medication, materials and disposables, patient nutrition and variable overheads) and fixed costs (fixed overheads, building, equipment, and furniture). All costs incurred by the hospital from admission to discharge were accounted for, which entails all pre-operative costs, the cost of surgery, and post-surgery costs until discharge. In addition, all complications that occurred after robotic colorectal surgery and prior to discharge were identified, as well as the costs of developing clinical pathways incurred by the multidisciplinary team of experts who designed them.

As a second step, for each cost item identified, the corresponding quantity of resources consumed was measured using different methods. Because resource consumption varies depending on the specific needs of each patient, data collected via the HMIS was used to measure it in the most precise way. Since it is used for costing purposes, this system tracks all resources consumed from admission to discharge, including materials and disposables; laboratory and diagnostic tests performed; all medication administered; nutrition; and all resources used to treat complications. The human resources dedicated to patients by all hospital staff involved were estimated using different methods. For the pre- and post-operative phases, estimates were obtained through a combination of direct observation and expert opinions. For the intra-operative phase, data collected from the HMIS were used to determine the time allocated to each patient. Missing cost items were measured through direct observation at the facility, physical counting (to document and count the type of equipment and furniture available in the department’s utilized areas for this intervention), secondary data (open source data on variable and fixed overhead expenses in German hospitals as a percentage of total costs) [24], and expert interviews with the hospital team (CP development).

As a third step, the cost of all measured items was valued by multiplying the resources consumed by their unit costs. To identify unit costs, a mixture of bottom-up and top-down approaches was relied upon (Table 1). Various data sources were used, including market prices in Euros (price year 2023), tariff agreements for estimating staff costs for healthcare personnel, scientific literature, and publicly accessible data from the German Federal Statistical Office.

For staff, the corresponding cost per minute was multiplied by the estimated minutes that each staff member spent in the continuum of care for each patient. For personal protective equipment, laboratory and diagnostics, medication, material and disposables, and nutrition, the unit cost was multiplied by the quantity used for each patient. For building infrastructure, the average commercial rental costs in that area of Germany were used to obtain the cost per square meter, which was then multiplied by the average size of a hospital room divided by the number of patients staying in that room, and the days of hospitalization of each patient. For equipment and furniture, the cost and useful life of each item were obtained, allowing the depreciated cost per day of each item to be determined, which was then multiplied with the utilization time corresponding to each patient. This included the surgical robot (Da Vinci SI, Intuitive Surgical, Inc.), which, according to the identified literature, was depreciated over a useful life span of 5 years [25–27]. It should be noted that the facility has a high volume of use of the robot, considering that it was used approximately four days per week with one surgery per day during the study period. If a maximum capacity utilization of five surgeries per week is considered, the robot’s capacity utilization is 80%. This is a crucial point in our analysis since the estimated unit cost per patient depends on the utilization capacity of the robot. To estimate fixed overheads, the robot maintenance costs were divided by the estimated number of surgeries in the facility to obtain the corresponding cost for each patient. Additionally, the percentage that fixed costs represent in German hospitals, according to open data sources (the official statistics of the German federal government, Statistisches Bundesamt - DESTATIS), was applied. Postoperative complications were classified according to the Clavien-Dindo grading system of surgical complications [28]. Complication-associated costs were calculated using a bottom-up micro-costing approach based on the resource consumption for the particular complications. Finally, to estimate the cost of developing the CP, the cost of staff time within the development team was used. Costs related to the development of CPs were included in the sensitivity analysis.

The methods used to measure and value all cost items, as well as the data sources are summarized in Table 1.

**Table 1:** Cost items and micro-costing methods

PERIOD	COST TYPE	ITEMS	MEASUREMENT	VALUATION	
			METHODS	METHODS	DATA SOURCE
PRE-OP	VARIABLE	CPs DEVELOPMENT	Expert interview	Bottom-up	Tariff agreement
PERI-OP	VARIABLE	STAFF	HMIS	Bottom-up	Tariff agreement
		PERSONAL PROTECTIVE EQUIPMENT	HMIS	Bottom-up	Market prices
		LABORATORY & DIAGNOSTICS	HMIS	Bottom-up	Market prices
		MEDICATION	HMIS	Bottom-up	Market prices
		MATERIAL & DISPOSABLES	HMIS	Bottom-up	Market prices
		NUTRITION	HMIS	Bottom-up	Market prices
		VARIABLE OVERHEADS	Secondary data	Top-down	Online - DESTATIS
	FIXED	FIXED OVERHEADS	Secondary data	Top-down	Scientific literature
		BUILDING	Direct observation	Bottom-up	Market prices
		EQUIPMENT & FURNITURE	Physical counting	Bottom-up	Market prices
POST-OP	VARIABLE	COMPLICATIONS*	HMIS	Bottom-up	Market prices

*Complications were limited to duration of inpatient stay

### Cost analysis

To compute and analyze costs, Microsoft Excel 2016 and Stata 16 were used. The total treatment cost for each of the 63 patients included in the study was calculated to derive the mean unit cost for both the CP group (*n* = 21; patients undergoing robotic colorectal surgery with a clinical pathway) and the no-CP group (*n* = 42; without a clinical pathway). In addition, the mean cost of individual items for each group was assessed to identify potential cost drivers. To compare total hospitalization costs between the two groups, an independent two-tailed t-test assuming unequal variances (Welch’s t-test) was performed, using a significance level of α = 0.05.

### Sensitivity analysis

A sensitivity analysis was conducted to assess uncertainty related to the hypothetical capacity utilization of the robotic system. Assuming that an operating room equipped for robotic surgeries is used up to five times a week, one robotic surgery per workday, the utilization capacity would be 100%. Approximately four robotic surgeries were performed per week during the study period, corresponding to a utilization rate of 80%, which reflects a setting with high utilization of the robotic system. However, other settings with less demand may operate at lower utilization rates. Therefore, the amortization cost of the robot was calculated over a five-year period using different utilization rates (20%, 40%, 60%, 80%, and 100%) to capture the differences that the volume of use of the robotic equipment would have on the estimated mean cost of colorectal robotic surgery in patients treated with and without CP. Development costs of the CP were not included in the base-case analysis but were incorporated in the sensitivity analysis to reflect diverse implementation scenarios. In addition, the sensitivity analysis included the development costs of the CP amortized over a five-year period, as well as a 3% discount rate, in accordance with values reported in the literature [29, 30].

## Results

Table 2 presents the baseline characteristics of the patients. The mean age was comparable between groups. A higher proportion of female and oncologic patients was observed in the no-CP group, whereas Crohn’s disease and diverticulitis were more prevalent in the CP group. A single case of benign tumor (adenoma) occurred in the no-CP group. Rectal resections were more frequently performed in the CP group, while colon resections were more common in the no-CP group. ASA physical status distribution was similar across both cohorts.

**Table 2:** Baseline patient data and surgical characteristics

Group	CP (*n* = 21)	no-CP (*n* = 42)
Age	63.1	63.5
Female sex	23.8% (5/21)	35.7% (15/42)
Diagnosis		
Carcinoma	71.4% (15/21)	85.7% (36/42)
Crohn’s Disease	14.2% (3/21)	2.4% (1/42)
Sigmoid Diverticulitis	14.2% (3/21)	9.5% (4/42)
Adenoma	0	2.4% (1/42)
Surgical Procedures		
Colon resection	23.8% (5/21)	35.7% (15/42)
Rectal resection	76.2% (16/21)	64.3% (27/42)
ASA		
I	0	5% (2/42)
II	33% (7/21)	40% (17/42)
III	66% (14/21)	54% (23/42)

Table 3 shows intra- and postoperative details. The mean length of stay (LOS) was longer in the CP group with 14.7 days compared to 13.2 days in the no-CP group. The surgery time (incision to suture) was also longer in the CP group with 387 min compared to 319 min in the no-CP group. When including anesthesiological care time, the mean procedural time was 547 min in the CP group, and 466 min in the no-CP group. For the CP group, mean anesthesiological care time was 160 min longer than the mean surgery time (387 min), whereas in the no-CP group, this difference was 147 min. This indicates that anesthesiological care took longer for the CP group. 11/42 (26%) patients in the no-CP group and 9/21 (43%) patients in the CP group had postoperative complications of any degree according to the Clavien-Dindo classification, with 6/42 (14%) and 2/21 (9%) complications grade ≥ 3, respectively.

**Table 3:** Summary of intra- and postoperative details

	CP (*n* = 21)	no-CP (*n* = 42)
Mean length of hospital stay (days)	14.7	13.2
Mean surgery time (min)		
Incision to suture	387	319
Including anesthesiological care	547	466
Complications	43% (9/21)	26% (11/42)
Major (≥ 3a)*	9% (2/21)	14% (6/42)
Minor (< 3)*	33% (7/21)	11% (5/42)

*Clavien-Dindo classification

The estimated mean cost per patient in the CP group was €12,663 (SD ± 2,944). The main cost drivers were material and disposables representing 33% of the mean cost, followed by staff with 20% of the total cost (Table 4). The estimated mean cost per patient in the no-CP group was €11,128 (SD ± 2,148). Here, the main cost drivers were also materials and disposables (33%) and staff (20%) of the total cost. The largest difference detected between the mean cost in the two groups was attributable to medications, with €1,003 in the CP group and €521 in the no-CP group.

The Welch’s t-test yielded a p-value of 0.113, indicating that the difference in total cost between groups was not statistically significant. The effect size observed, Cohen’s d = 0.46, suggests that the magnitude of the difference between the two groups is moderate.

**Table 4:** Cost per item in the two groups

Cost Type	Items	Mean cost per group (€)			Proportion per group (%)	
		CP	no-CP	CP		no-CP
Variable	Staff	2,584	2,180	20.4		19.6
	Personal Protective Equipment	12	12	0.1		0.1
	Laboratory + Diagnostics	648	529	5.1		4.8
	Medication	1,003	521	7.9		4.7
	Material + Disposables	4,179	4,174	33.0		37.5
	Nutrition	191	172	1.5		1.5
	Complications (minor & major)	476	121	3.8		1.1
Fixed	Building	174	160	1.4		1.4
	Equipment + Furniture	1,568	1,566	12.4		14.1
	Overhead (fix + variable)	1,827	1,692	14.4		15.2
**Estimated mean cost**		**12**,**663**	**11**,**128**	**100.0**		**100.0**
**T-test (Estimated Mean Costs)**						
	**Standard Deviation**	**± 2**,**944**	**± 2**,**148**			
	**P-Value**	**0.113**				
	**Cohen’s d**	**0.46**				

The sensitivity analysis shows variability in the hypothetical capacity utilization of the robot with different utilization percentages ranging from 20% to 100% (Table 5). The estimated costs of robotic colorectal surgery in patients treated with CP include the cost of development of CP distributed over a period of five years at different capacity utilization. Development costs were calculated based on the hourly rate of the surgeon who developed them multiplied by the estimated development time of 150 h. Moreover, 30 h per year were allocated to update the CP as needed. Additionally, these estimates also include a discount rate of 3%.

**Table 5:** Sensitivity analysis at different capacity utilization of the robotic equipment

Da Vinci Capacity Utilization (%)	Y1 (€)	Y2 (€)	Y3 (€)	Y4 (€)	Y5 (€)
100	12,364	12,752	13,151	13,562	13,986
80	12,673	13,074	13,487	13,912	14,350
60	13,197	13,622	14,060	14,510	14,974
40	14,284	14,756	15,242	15,741	16,256
20	17,883	18,513	19,161	19,830	20,519

**Fig. 1:**
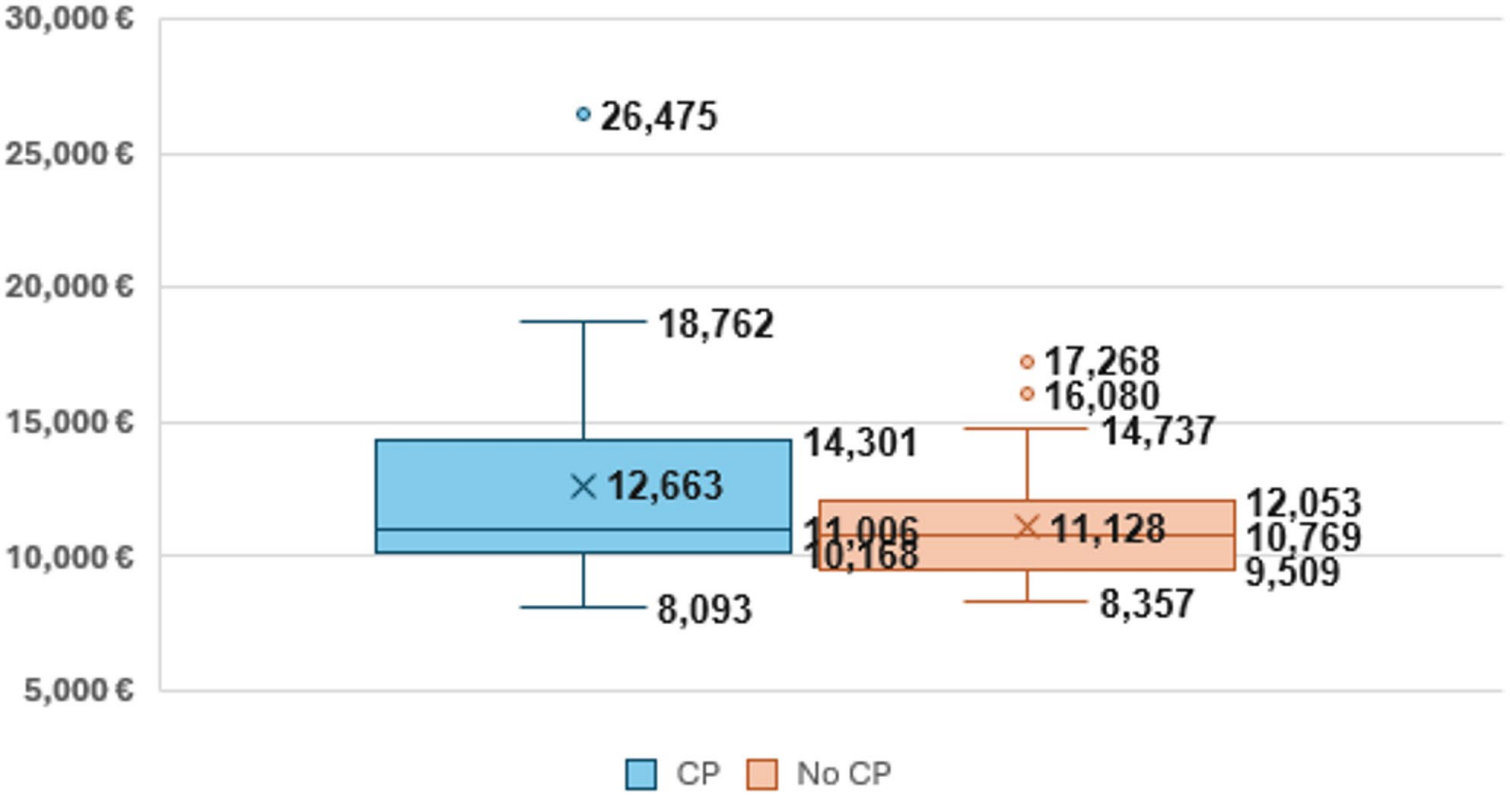
Cost variation per group including complications

Figure 1 shows the cost variation between individual patients in the two groups. In the CP group, the interquartile range was from €10,168 to €14,301, with a minimum cost of €8,093 and a maximum cost of €26,475. In the no-CP group, the interquartile range was from €9,509 to €12,053, with a minimum cost of €8,357 and a maximum cost of €17,268.

## Discussion

At the time of writing this article, no other studies were identified that estimate the effect of treating patients with a CP for robotic colorectal surgery on treatment cost. By applying a bottom-up costing approach, we provide granular insight into resource utilization and cost drivers in a real-world tertiary care setting. Our results provide evidence to facilitate informed decision-making regarding the adoption of CPs for robotic colorectal resections. While our findings suggest that implementing this specific CP for robotic colorectal surgery was not associated with cost savings, the effect size observed (Cohen’s d = 0.46) suggests a moderate difference in mean cost between the intervention and control groups. However, this difference did not reach statistical significance, and given the exploratory nature of the study and its methodological constraints, these findings should be interpreted with caution and not as evidence of a causal relationship. Further evaluation of the CP’s impact on clinical outcomes is necessary to determine whether any potential improvements in patient care may offset the higher costs. In addition, a key strength of this study lies in the use of the micro-costing methodology employed, which could serve as a template for future micro-costing analyses concerning the integration of CPs in robotic surgery.

Based on our estimates, it was found that the primary cost drivers in both groups were material and disposables, accounting for 33% and 37.5% of total costs, respectively. This was predominantly driven by expenses related to the robotic surgery instruments and disposables. Personnel costs followed, comprising 20.4% of the estimated costs in the CP group and 19.6% in the no-CP group. Surgery stands out as the most expensive aspect due to the time resources spent by each team member during the operative procedure. Equipment represents the third relevant cost driver, constituting 12.4% and 14.1% of the estimated expenses in the intervention and the no-CP groups, respectively. The primary cost within this category is attributed to the robotic equipment’s allocation per patient. Furthermore, in our sensitivity analysis at different capacity levels of the robotic equipment, it can be concluded that the higher the utilization, the lower the estimated mean cost per patient.

Our findings indicate that the mean estimated cost of perioperative care was higher in patients undergoing robotic colorectal surgery with CPs than in the no-CP group. This disparity primarily stemmed from the CP group’s longer surgery duration, which resulted in increased costs due to the extended utilization of healthcare professionals’ time. Although surgical CPs aim to standardize all perioperative processes, the absolute duration of the operation (i.e., the incision-to-suture time) can probably not be modified in a relevant manner unless the CP stipulates dedicated steps of the operation, which was not the case in our setting. One crucial factor to consider regarding incision-to-suture time and clinical outcomes is the learning curve in robotic surgery. Studies on robotic-assisted colorectal surgery suggest that proficiency is achieved somewhere between 15 and 30 cases [31–34]. The study by Jung-Park (2014), involving 130 consecutive patients who underwent robotic low anterior resection, suggests three phases in the learning curve of robotic colorectal surgery: (1) initial period, (2) competent period, and (3) challenging period. In that study, technical competence required to achieve feasible perioperative outcomes is reached only after 79 surgeries [35]. During the course of our study, there were changes in surgical staff performing robotic surgery, affecting both the CP group and the no-CP group. As a result, we believe that learning curve effects might have influenced results. It cannot be excluded that this may have contributed to the disparity in surgical duration and the associated costs observed between the two groups. Another determining factor was the cost of medication administered to patients in the CP group. This was partially driven by the higher incidence of complications in the CP group, which led to more medication being used and to a longer hospital stay. It is also worth highlighting the disparity between the two groups in the time difference between anesthesiological care and the actual incision-to-suture time. The CP aimed to standardize processes during preparation for surgery and anesthesia induction. Yet, this did not result in shorter anesthesia care times. The reasons for this could not be assessed in detail in our analyses.

The CP group experienced a higher incidence of minor and major complications. One of the declared aims of CP is to reduce the incidence of complications by standardizing and thus improving perioperative processes [36, 37]. It must be acknowledged that in this specific use case, this aim was not reached. These findings are in line with previous studies in colorectal surgery, which could also not demonstrate reduced postoperative morbidity after CP implementation [38, 39], whereas other studies showed pertinent success in reducing morbidity [40–42]. The conditions under which a CP can be successful in that regard are probably highly specific to the treatment setting and the specific contents of the CP, which suggests that CPs require continuous refinement and readjustment to achieve their aims. In our case, the higher incidence of complications in the CP group led to elevated expenses associated with medication, additional interventions and prolonged hospital stays. These factors contributed to increased costs across various categories including building, staff, nutrition, and overheads.

Length of hospital stay was rather high in both groups compared to national and international benchmarks [43, 44]. Complications and the associated longer inpatient treatment time might have played a role, which is also reflected in the higher length of stay in the CP group, in which proportionally more patients suffered complications. Other contributing factors might be obstacles towards fast discharge or towards transfer to other facilities such as rehabilitation facilities due to organisational problems.

We acknowledge that the primary limitation of our study lies in the small sample size, comprising 21 patients in the CP group and 42 patients in the no-CP group. Patients were included consecutively before and after CP implementation, and no randomization was performed. Therefore, differences in case mix between groups may have influenced the observed cost differences but may also have led to a more accurate reflection of ‘true’ costs according to the intention-to-treat principle due to the inclusion of individual high-cost cases. In our study protocol, we intended to include two groups of 45 patients each. However, hospital restrictions brought on by the COVID-19 pandemic led to many operations being postponed or not performed robotically, preventing us from reaching the planned number of patients in the CP group. Moreover, the temporal separation between study groups introduces an important source of potential confounding. Patients in the no-CP group were treated predominantly before the COVID-19 pandemic, whereas patients in the CP group were treated during the pandemic period. System-level changes during COVID-19, including altered hospital workflows, increased infection control measures, and potential shifts in patient selection, may have influenced both resource utilization and clinical outcomes independently of CP implementation. Furthermore, the study was conducted at a single tertiary care hospital in Germany. Consequently, the generalizability of the results may be limited, even within the German health care system, where the treatment landscape is heterogeneous and relevant differences exist in how care is structured across facilities offering robotic colorectal surgery. This limits the direct transferability of the results to other institutions. Another limitation is posed by the baseline differences between groups, including variation in diagnoses and types of colorectal surgery, which may have affected comparability. Given the relatively small sample size, formal statistical comparisons of baseline characteristics were not performed, as such analyses would have had limited interpretability and statistical power. Therefore, perioperative treatment characteristics across these procedures are somewhat heterogeneous. Although a range of colorectal procedures was included, colon and rectal resections share comparable perioperative pathways and resource utilization patterns, which supports their joint analysis within a unified costing framework. All patients followed a comparable perioperative care pathway and were analyzed within a unified costing framework; however, differences in surgical complexity and resource requirements may have influenced cost estimates.

It is also important to emphasize that this study represents a cost analysis rather than a full economic evaluation, as cost-effectiveness cannot be determined without integrating clinical outcomes. Complementary analyses incorporating these outcomes are currently ongoing and will be essential to enable a full economic evaluation of CP implementation in robotic colorectal surgery in the given setting. Therefore, the present findings should be interpreted as a detailed description of cost structures and drivers rather than a definitive assessment of the value of CP implementation. Given the small sample size, the non-randomized design with a historical comparison, the temporal separation between groups, and the presence of baseline differences, a possible causal inference regarding the effect of CP implementation on costs remains limited. Accordingly, our results should be considered exploratory and hypothesis-generating rather than definitive. While the study provides detailed insight into cost structures and potential cost drivers in robotic colorectal surgery, further research based on larger, prospectively designed, and ideally randomized studies is required to confirm these findings and more robustly assess the economic impact of CP implementation in robotic colorectal surgery.

Despite these limitations, our study offers valuable insights into the economic structure of robotic colorectal surgery and highlights key factors influencing costs. To our knowledge, detailed micro-costing analyses of CPs in robotic colorectal surgery remain scarce, and the present study provides granular insight into cost structures that are not captured in aggregate economic evaluations. Such information is essential for informing hospital-level decision-making and for designing future economic evaluations. Future research should aim to combine detailed cost data with clinical outcomes in larger, prospective multicenter studies with concurrent comparison groups, allowing for a more robust assessment of the cost-effectiveness of clinical pathways in robotic colorectal surgery and help to better isolate the effect of CP implementation from other contextual factors. Notwithstanding these findings, it is important to emphasize that the use of CPs for perioperative care in robotic colorectal surgery has helped standardize procedures and clearly define the tasks of all personnel involved. A qualitative perspective on hospital staff perceptions could provide insight into the reasons behind our results; however, this is beyond the scope of our study.

## Conclusion

The use of a CP in robotic colorectal surgery did not reduce perioperative costs compared to the group treated without a CP, with staff and material costs representing the main cost drivers in both groups. From an economic perspective, no statistically significant reduction in perioperative costs was observed with the implementation of a CP for robotic colorectal surgery in its current form. These findings should be interpreted cautiously, given the exploratory design, potential confounding, and absence of integrated clinical outcome data.

## Supplementary Information

Below is the link to the electronic supplementary material.

Supplementary Material 1

Supplementary Material 2

## Data Availability

The datasets used and/or analysed during the current study are available from the corresponding author on reasonable request.

## Abbreviations

CP: Clinical Pathway
HMIS: Health Management Information System
LOS: Length of Stay
