# Supplementary material for: A cost comparison between patients undergoing robotic colorectal surgery with and without a clinical pathway
Source: Cost Eff Resour Alloc. 2026 May 23;24:66. doi: 10.1186/s12962-026-00770-9 (PMC13198061; doi:10.1186/s12962-026-00770-9)
Supplement: Supplementary file 2 — Supplementary Material 2 [file 12962_2026_770_MOESM2_ESM.pdf]

NAME:	FIRST NAME:	DATE OF BIRTH:	DATE OF SURGERY:	DISCHARGE DATE:

**Rectal resection with protective ileostomy**	**Before admission**	**INPATIENT PHASE Day of admission**	**Day of operation**	**Postop day 1**	**Postop day 2**	**Postop day 3-6**	**DISCHARGE Postop day 7**
**DIAGNOSTICS / MONITORING**	☐ History<br>☐ Clinical assessment with digital examination<br>☐ Lab values (hemogram, “internal order set”, coagulation, HbA1c in diabetics, CEA)<br>☐ Full colonoscopy, biopsy where indicated<br>☐ Pelvic MRI<br>☐ Thoracoabdominal CT<br>☐ *Multidisciplinary tumor board after completion of staging: Neoadj. Therapy?*<br>☐ *If neoadj. therapy indicated readmission after completion*<br>☐ *After long regime RCTx, MRI for Regression, Thoracoabdominal CT for controlling metastases*<br>☐ Anesthesiologic assessment (including consent for epidural), if needed further exams (e.g. cardiological assessment)<br>☐ Appointment of admission / surgery	☐ Basic lab values (hemogram, “basic order set”, coagulation, HbA1c in diabetics, CEA, if no recent values available<br>☐ Prepare two units of packed red blood cells<br>☐ Compile all relevant imaging	*Intraoperative*<br>☐ Monitor vital signs (Blood pressure, heart rate, body temperature (>36°C), FiO_2 (0,7), CVP if CVC was placed)<br>☐ Monitor relaxation<br>☐ Glucose (120 – 160 mg/dl)<br>*Postoperative*<br>☐ Surgeon decides ICU/normal ward<br>☐ Monitor vital signs<br>☐ Monitor diuresis<br>☐ Monitor drainage fluid (if drainage in place)<br>☐ At night: hemogram, „basic order set“, coagulation<br>☐ Chest x-ray if CVC was placed<br>☐ Check dressings<br>☐ Check epidural line	☐ Monitor vital signs every 8 hours<br>☐ Monitor drainage fluid (if drainage in place)<br>☐ Hemogram, „basic order set“, CRP	☐ Monitor vital signs every 12 hours<br>☐ Monitor drainage fluid (if drainage in place)<br>☐ Hemogram, „basic order set“, CRP	☐ Monitor vital signs every 12 hours<br>☐ Monitor drainage fluid (if drainage in place)<br>☐ Hemogram, „basic order set“, CRP (POD 4 and 6)<br>☐ *Discuss further treatment at multidiscplinary tumor board once histology available*	☐ Monitor vital signs in the morning
**ANAESTHESIA**			☐ Cefuroxim 1,5gr i.v. / Metronidazol 500 mg i.v. <u>30-60 min before incision</u> (if Penicillin allergy: Clindamycin 900 mg + gentamycin 5 mg / kg i.v<br>☐ Prewarming<br>☐ Intubation				
**Catheters**			☐ G16 venous cannula<br>☐ CVC<br>☐ Arterial line in high-risk patients<br>☐ Place gastric tube intraoperatively, remove upon extubation<br>☐ Thoracic epidural line (Th 8-10)	☐ Remove arterial line if placed	☐ Remove CVC if placed<br>☐ Remove venous line		
**Urinary catheter**			☐ Foley after induction of anesthesia			☐ Remove foley after epidural removal	
**Intraop position**			☐ Supine position, vacuum mattress, Stirrups with lower 30°, bilateral arm tuck, chest and pelvic fastening, abdominal and perineal skin prep				
**Drains**			☐ Intrapelvic drainage<br>☐ Surgeon decides the type of drainage			☐ Remove drainage if in place and secretion normal (POD 6)	

**Nutrition**	☐ No restriction, encourage sufficient intake	☐ Liquids only (Mechanical bowel preparation)	☐ ~~Sweetened tea until~~ 2 hours preop (6 am if no exact time of operation known)Start tea 2 hours postop (max 1500 ml); 2 cups of yoghurt	☐ No restriction (specific diet for diabetics)<br>☐ Drink >1500 ml	☐ No restriction (specific diet for diabetics)<br>☐ Drink >1500 ml	☐ No restriction (specific diet for diabetics)<br>☐ Drink >1500 ml	☐ No restriction (specific diet for diabetics)<br>☐ Drink >1500 ml
**INFUSIONS**			☐ Aim at intraoperative normovolemia<br>☐ Postop isotonic electrolyte solution (saline in patients with renal failure < 500 ml i.v.)<br>☐ if postop nausea / vomiting more infusion	☐ none	☐ none	☐ none	☐ none
**STOOL/BOWEL PREPARATION**		☐ Mechanical bowel preparation + oral antibiotics	☐ Preop enema	☐ Magnesium oxide solution tid until first stool passed	☐ Magnesium oxide solution tid until first stool passed	☐ Magnesium oxide solution tid until first stool passed	☐ Magnesium oxide solution tid until first stool passed
**MEDICATION**	☐ Continue previous drug regimen<br>☐ Stop vitamin K antagonists, replace with nadroparin 0,1 ml / 10 kg bodyweight bid.<br>☐ Plan to stop oral antidiabetics on day of admission	☐ Continue previous drug regime with described exceptions<br>☐ Insulin scheme: glucose 140-200 mg/dl: 4 iU; 200-280 mg/dl: 8 iU; > 280 mg/dl 12 iU rapid acting insulin sc, check glucose level after 2 hours<br>☐ Nadroparin 0,1 ml / 10 kg bodyweight bid	☐ Continue previous drug regimen with described exceptions<br>☐ Insulin scheme: glucose 140-200 mg/dl: 4 iU; 200-280 mg/dl: 8 iU; > 280 mg/dl 12 iU rapid acting insulin sc, check glucose level after 2 hours<br>☐ Premedication as ordered by anesthetists<br>☐ Nadroparin 0,3 ml sc at night (or weight adapted dose if full anticoagulation) Metoclopramid 10mg 1-1-1, if required Ondansetron 8mg i.v. for PONV prophylaxis	☐ Continue previous drug regimen with described exceptions<br>☐ Insulin scheme: glucose 140-200 mg/dl: 4 iU; 200-280 mg/dl: 8 iU; > 280 mg/dl 12 iU rapid acting insulin sc, check glucose level after 2 hours<br>☐ Pantoprazol 40 mg po 0-0-1<br>☐ Nadroparin 0,3 ml sc at night (or weight adapted dose bid if full anticoagulation)<br>☐ Metoclopramid 10mg 1-1-1, if required Ondansetron 8mg i.v. for PONV prophylaxis	☐ Continue previous drug regimen with described exceptions<br>☐ Insulin scheme: glucose 140-200 mg/dl: 4 iU; 200-280 mg/dl: 8 iU; > 280 mg/dl 12 iU rapid acting insulin sc, check glucose level after 2 hours<br>☐ Pantoprazol 40 mg po 0-0-1<br>☐ Nadroparin 0,3 ml sc at night (or weight adapted dose bid if full anticoagulation)	☐ Continue previous drug regimen with described exceptions<br>☐ Start with oral antidiabetics on 3. postop. day<br>☐ Insulin scheme: glucose 140-200 mg/dl: 4 iU; 200-280 mg/dl: 8 iU; > 280 mg/dl 12 iU rapid acting insulin sc, check glucose level after 2 hours<br>☐ Pantoprazol 40 mg po 0-0-1<br>☐ Nadroparin 0,3 ml sc at night (or weight adapted dose bid if full anticoagulation)	☐ Continue previous drug regimen with described exceptions<br>☐ Pantoprazol 40 mg po 0-0-1<br>☐ Nadroparin 0,3 ml sc at night (or weight adapted dose bid if full anticoagulation)<br>☐ Plan to start with oral anticoagulation after postop. 14 days
**TRANSFUSIONS**			☐ only if hb < 8 mg/% or cardiopulmonary instability	☐ only if hb < 8 mg/% or cardiopulmonary instability	☐ only if hb < 8 mg/% or cardiopulmonary instability	☐ only if hb < 8 mg/% or cardiopulmonary instability	
**ANALGESIA**<br><br>**intravenous**			With or without epidural line:<br>☐ Metamizol 1g or Paracetamol 1g 1-1-1-1	With or without epidural line:<br>☐ Metamizol 1g or Paracetamol 1g 1-1-1-1	☐ avoid	☐ avoid	

**Oral**			Without epidural line:<br>☐ Oxycodone long acting 10mg / 20mg<br>if VAS ≥ 4 add Oxycodone 5mg / 10mg quick acting	Without epidural line:<br>☐ Oxycodone long acting 10mg / 20mg<br>☐ if VAS ≥ 4 add Oxycodone 5mg / 10mg quick acting	With or without epidural line:<br>☐ Metamizol 1g or Paracetamol 1g if needed max. 4g<br>Without epidural line:<br>☐ Oxycodone long acting 10mg / 20mg<br>☐ if VAS ≥ 4 add Oxycodone 5mg / 10mg quick acting<br>☐ try to reduce doses of Oxycodone on postop day 3	☐ Metamizol 1g or Paracetamol 1g if needed max. 4g<br>☐ avoid systemic opioids<br>☐ Oxycodone 5mg / 10mg quick acting if needed	☐ Metamizol 1g or Paracetamol 1g if needed max. 4g<br>☐ avoid systemic opioids<br>☐ on discharge prescribe Metamizol 1g or Paracetamol 1g 1-1-1-1 if needed
**Epidural line**			☐ Thoracic epidural line (Th 8-10) with continous infusion: Ropivacain 0,18% + Sufentanil 0,45 µg/ml (200 ml Ropivacain + 20 ml sufentanil (50 µg / 10ml) = 220ml, flow 4-6 ml/h patient bolus 2 ml with an interval 20 min	☐ Thoracic epidural line with continous infusion (see before)	☐ Only patient bolus 4 ml with an interval 20 min	☐ remove on postop day 3 (anticoagulation after 4h)	
**Postop care**						☐ *Apply for postop rehab treatment if no adjuvant therapy planned*	
**QUALITY CONTROL / DOCUMENTATION / DRG**			☐ Verify correct shipment of histological samples (responsible: surgeon)<br>☐ Write and print brief report of operation and postop orders (surgeon)<br>☐ Dictate report of operation (surgeon)<br>☐ Code performed procedure (surgeon)			☐ Check histology<br>☐ Fix aftercare appointment<br>☐ Prepare discharge report	☐ Hand discharge report to patient; forward to consultant for signature<br>☐ DRG coding
**PATIENT INFORMATION / EDUCATION**	☐ Recommend smoking and drinking cessation 14 days prior to surgery<br>☐ Inform patient and relatives about planned surgery and principles of enhanced recovery scheme (information leaflet)	☐ Informed consent<br>☐ Re-emphasize principles of enhanced recovery scheme (information leaflet)<br>☐ Ask which contacts to inform directly postop<br>☐ Recruit in clinical studies where applicable	☐ Call designated contacts (surgeon)<br>☐ Call referring physician (surgeon)<br>☐ Inform patient once fully awake				☐ Discharge information, communicate histology and further treatment recommendation if already available<br>☐ Call referring physician
**NURSING**<br><br>**Admission / discharge**		☐ Welcome and inform patient<br>☐ Nursing history	*Postoperative:*<br>☐ Inform patient<br>☐ Copy postoperative orders into daily chart			☐ Prepare discharge documents, fix relevant appointments<br>☐ Provide discharge information	☐ Provide discharge information
**Ward rounds**		☐ Participate in rounds<br>☐ Copy orders into daily chart<br>☐ Insert lab sheets into daily charts	☐ Participate in rounds<br>☐ Copy orders into daily chart<br>☐ Insert lab sheets into daily charts	☐ Participate in rounds<br>☐ Copy orders into daily chart<br>☐ Insert lab sheets into daily charts	☐ Participate in rounds<br>☐ Copy orders into daily chart<br>☐ Insert lab sheets into daily charts	☐ Participate in rounds<br>☐ Copy orders into daily chart	☐ Participate in rounds<br>☐ Copy orders into daily chart<br>☐ Insert lab sheets into daily charts

**Documentation**	☐ Insert CP sheet into patient file	☐ Insert CP sheet into daily chart	☐ Document DRG codes relating to nursing activities<br>☐ Document nursing activities in daily chart	☐ Document DRG codes relating to nursing activities<br>☐ Document nursing activities in daily chart	☐ Document DRG codes relating to nursing activities<br>☐ Document nursing activities in daily chart	☐ Document DRG codes relating to nursing activities<br>☐ Document nursing activities in daily chart	☐ Document DRG codes relating to nursing activities<br>☐ Document nursing activities in daily chart
**Patient care**		☐ Personal care according to nursing plan<br>☐ Prepare drugs, assist in intake if needed	☐ Personal care according to nursing plan<br>☐ Prepare drugs, assist in intake if needed	☐ Personal care according to nursing plan<br>☐ Prepare drugs, assist in intake if needed<br>☐ Pneumonia prophylaxis (incentive spirometry, mucolysis, patient education)<br>☐ Mechanical DVT prophylaxis	☐ Personal care according to nursing plan<br>☐ Prepare drugs, assist in intake if needed<br>☐ Pneumonia prophylaxis (incentive spirometry, mucolysis, patient education)<br>☐ Mechanical DVT prophylaxis	☐ Personal care according to nursing plan<br>☐ Prepare drugs, assist in intake if needed<br>☐ Pneumonia prophylaxis (incentive spirometry, mucolysis, patient education)<br>☐ Mechanical DVT prophylaxis	☐ Personal care according to nursing plan<br>☐ Prepare drugs, assist in intake if needed<br>☐ Pneumonia prophylaxis (incentive spirometry, mucolysis, patient education)<br>☐ Mechanical DVT prophylaxis
**Mobilisation / physiotherapy**			☐ 5 hours postop: walk on aisle, mobilise in armchair for 2 hours,	☐ Walk on aisle at least twice, mobilise out of bed for >8 hours<br>☐ Physiotherapy in patients with COPD, walking impairment, bedridden patients	☐ Walk on aisle at least twice, in bed only for afternoon nap and at night<br>☐ Physiotherapy in patients with COPD, walking impairment, bedridden patients	☐ Walk on aisle at least twice, in bed only for afternoon nap and at night<br>☐ Physiotherapy in patients with COPD, walking impairment, bedridden patients	☐ Walk on aisle at least twice, in bed only for afternoon nap and at night
**Patient checks**		☐ Vital signs (heart rate, blood pressure, temperature)<br>☐ VAS for pain (1-10)	☐ Vital signs (heart rate, blood pressure, temperature)<br>☐ VAS for pain (1-10)	☐ Vital signs (heart rate, blood pressure, temperature)<br>☐ VAS for pain (1-10)	☐ Vital signs (heart rate, blood pressure, temperature)<br>☐ VAS for pain (1-10)	☐ Vital signs (heart rate, blood pressure, temperature)<br>☐ VAS for pain (1-10)	☐ Vital signs (heart rate, blood pressure, temperature)<br>☐ VAS for pain (1-10)
**Wounds / drainages / ostomy**			☐ Check dressings<br>☐ Ostomy care	☐ Check dressings<br>☐ Ostomy care	☐ Change dressings<br>☐ Ostomy care	☐ Change dressings<br>☐ Ostomy care education (POD 3)<br>☐ Remove drainage upon physician order (POD 6)	☐ Change dressings<br>☐ Ostomy care

**Robotic rectal resection**	**Before admission**	**INPATIENT PHASE**<br>**Day of admission**	**Day of operation**	**Postop day 1**	**Postop day 2**	**Postop day 3-6**	**DISCHARGE**<br>**Postop day 7**
Deviations<br>*(please document with day, type of deviation, and reason)*	Deviation:<br>Reason:<br>Deviation:<br>Reason:<br>Deviation:<br>Reason:<br>Deviation:<br>Reason:<br>Deviation:<br>Reason:<br>Deviation:<br>Reason:<br>Deviation:<br>Reason:	Deviation:<br>Reason:<br>Deviation:<br>Reason:<br>Deviation:<br>Reason:<br>Deviation:<br>Reason:<br>Deviation:<br>Reason:<br>Deviation:<br>Reason:<br>Deviation:<br>Reason:	Deviation:<br>Reason:<br>Deviation:<br>Reason:<br>Deviation:<br>Reason:<br>Deviation:<br>Reason:<br>Deviation:<br>Reason:<br>Deviation:<br>Reason:<br>Deviation:<br>Reason:	Deviation:<br>Reason:<br>Deviation:<br>Reason:<br>Deviation:<br>Reason:<br>Deviation:<br>Reason:<br>Deviation:<br>Reason:<br>Deviation:<br>Reason:<br>Deviation:<br>Reason:	Deviation:<br>Reason:<br>Deviation:<br>Reason:<br>Deviation:<br>Reason:<br>Deviation:<br>Reason:<br>Deviation:<br>Reason:<br>Deviation:<br>Reason:<br>Deviation:<br>Reason:	Deviation:<br>Reason:<br>Deviation:<br>Reason:<br>Deviation:<br>Reason:<br>Deviation:<br>Reason:<br>Deviation:<br>Reason:<br>Deviation:<br>Reason:<br>Deviation:<br>Reason:	Deviation:<br>Reason:<br>Deviation:<br>Reason:<br>Deviation:<br>Reason:<br>Deviation:<br>Reason:<br>Deviation:<br>Reason:<br>Deviation:<br>Reason:<br>Deviation:<br>Reason:
